# The synergistic interaction of systemic inflammation, dysbiosis and antimicrobial resistance promotes growth restriction in children with acute severe malnutrition: An emphasis on *Escherichia coli*

**DOI:** 10.3389/frabi.2022.1001717

**Published:** 2022-10-24

**Authors:** Rene Arredondo-Hernandez, Christina Siebe, Gonzalo Castillo-Rojas, Samuel Ponce de León, Yolanda López-Vidal

**Affiliations:** ^1^ Laboratorio de Microbioma, División de Investigación y División de Posgrado, Facultad de Medicina, Universidad Nacional Autónoma de México, Mexico City, Mexico; ^2^ Instituto de Geología, Universidad Nacional Autónoma de México, Mexico City, Mexico; ^3^ Programa de Inmunología Molecular Microbiana, Departamento de Microbiología y Parasitología, Facultad de Medicina, Universidad Nacional Autónoma de México, Mexico City, Mexico

**Keywords:** severe acute malnutrition, *Escherichia coli*, gut inflammation, microbiota dysbiosis, antimicrobial resistance, growth restriction

## Abstract

A healthy development is denied to millions of children worldwide as harsh life conditions manifest themselves in an altered inflammation-prone microbiome crosstalk environment. Keynote of this tragedy is that insufficient nutritious amino acid blocks lipids-intake to sustain diverse microbiota, and promotes the generalist strategy followed by *Escherichia coli* -besides other proteobacteria- of shifting gut metabolism, subverting the site specificity of first immune reaction. Furthermore, it could be hypothesized that selective success lies in their ability to induce inflammation, since this phenomenon also fuels horizontal gene transfer (HGT). In this review, we dilucidate how immune mechanisms of environmental enteric dysfunction affect overgrowth restriction, infectious morbidity rate, and acquired lifelong risks among severe acute malnourished children. Also, despite acknowledging complexities of antimicrobial resistant enrichment, we explore and speculate over the links between virulence regulation and HGT as an indissociable part in the quest for new inflammatory niches by open genome bacteria, particularly when both collide in the most vulnerable.

## Introduction

Severe acute malnutrition (SAM) affects over 28 million children worldwide, mostly in underdeveloped countries, and every year 1-2 million children die due to this preventable condition (Collins et al., 2006; Ghimire et al., 2020; Moyer et al., 2020). Among other disorders, these children are at an increased risk of morbidity and mortality from infectious diseases, further compromising full development and healthy life expectancy. Since they are frequently found in households with polluted water, crowding, and inconsistent income sources, the likelihood of being affected by any potentially pathogenic microorganism, and therefore of undergoing severe infections leading to fatal consequences, is high for these children (van Cooten et al., 2019). Importantly, due to the marginality associated with SAM, its pathogenesis and the mechanisms underlying the increased risk and negative outcomes from infection in SAM children, are not well defined.

Previous reports indicate that children with SAM are characterized by an intense basal inflammation starting at the gut, which begins with acute diarrheic events that eventually become chronic (Bhatnagar et al., 2019; Jain et al., 2020). Additionally, they suffer from high circulating bacterial lipopolysaccharide (LPS) levels, markers of intestinal damage, histological enteropathy, and an amplified whole blood inflammatory response to LPS (Amadi et al., 2017). Metabolic changes in children with SAM lead to reduced levels of short-chain fatty acids (SCFA), which induces a loop of a constant pro-inflammatory state, impairing their normal intestinal function (Attia et al., 2016). In addition, it has been shown that the gut microbiota composition of SAM children is dramatically different and less diverse from that of children with a normal dietary status (Tidjani Alou et al., 2017). Instead of ensuring a homeostatic microenvironment in the gut, the dysbiotic microbiota not only potentiates the inflammation in affected children, but also dramatically limits the synthesis and absorption of nutrients at the gut coming from the already scarce diet in SAM children, confining their full growth and development in a critical period of their lives (Calder et al., 2021). The whole inflammation-dysbiosis-growth restriction phenomena in SAM children could be attributed in part to the constant ingestion of fecal-polluted water and alimentary products coming from contaminated soil enriched in enteropathogenic bacteria such as *Escherichia coli* (Odonkor and Mahami, 2020), which are prone to promiscuous horizontal transference of antimicrobial resistant genes (Frazão et al., 2019).

In this review, we will cover the subjects of antimicrobial resistance (AMR), microbiome and inflammation in malnourished children, with a central thread in *E. coli* as part of feedback loop reinforcing a constant state of inflammation through undernutrition, and establishing a metabolic environment which in turn allows the acquisition of AMR genes, whereas it also limits normal growth and development of malnourished children.

## Severe acute malnutrition in children; perpetuation, burden, and health consequences at the marginality

Controlled trails and omics technologies are clarifying major misconceptions around the actual burden of enteric bacteria infection. It comes to light that the most pervasive effects over health take place along stretch-time windows of development. It appears that through dysfunctional microbiome crosstalk, immunometabolic reprogramming results in growth stunt and cognitive impairment in childhood, whereas infection, even when asymptomatic, also adds to other exposures, leading to chronic disease later in life.

Uncontrovertibly, in children from low-income settings, interindividual immune function against bacteria varies, likely as mother´s inflammation during pregnancy and recurrent pathogen-associated molecular patterns (PAMPs) exposure does, due to environmental enteropathy in severe acute malnutrition children living in close contact with enteric bacteria inside their household.

Severe Acute Malnutrition (SAM) is a critical public health problem, and estimates suggest it affects more than 28 million children worldwide, especially in low-income households in South Asia and Sub-Saharan Africa. This condition dramatically predisposes children to increased rates of morbimortality, particularly due to infectious diseases. In fact, over the past 70 years, case-fatality rates of hospitalized SAM children in underdeveloped countries have been maintained at 20-30% (Collins et al., 2006), and up to 7.4-7.8% of all child deaths are associated to SAM (Bartels et al., 2019). Furthermore, SAM is linked to negative economic consequences due to loss of life-productivity and to high costs for the health system (Moyer et al., 2020), which can rise up to US$453 per child in their ambulatory community-based treatment alone (Bachmann, 2010).

### SAM epidemiology and risk factors

SAM is remarkably relevant in the pediatric population during their first 5 years of life, since this is the critical period for development and fast physical growth in children (Ghimire et al., 2020). According to the World Health Organization (WHO), SAM could be clinically identified as a mid-upper arm circumference measurement <115 mm, or by the weight adjusted to height >3 standard deviation Z-scores below the median within this range of age (Bhadoria et al., 2017; Ghimire et al., 2020). Alternatively, SAM can be diagnosed in children with a weight-for-height measurement of ≤70% than the expected median, and in those showing bilateral pitting edema (Collins et al., 2006). However, due to inconsistent measurement cutoffs that may vary by country, and mostly to the lack of public attention, SAM has been historically underdiagnosed (Moyer et al., 2020). A multicentric study covering 15,060 children of 6 to 59 months old in the Democratic Republic of the Congo, Senegal and Nepal, showed a prevalence of SAM in 4.7% of them, and, importantly, 5% of the evaluated population died during the course of the study in 2019 (Schwinger et al., 2019). In India, by 2017, a national prevalence was estimated of 7.9% in children from 6 to 60 months old, but in a specific rural region located at the north of the country, it was found only in 2.2% of the 18,463 enrolled children (Bhadoria et al., 2017). In Mexico, a 2014 cross-sectional study covering 763 children hospitalized in a public hospital over 9 months, found that 6.2% of preschool children had SAM (Muñoz-Esparza et al., 2018).

SAM children lack an adequate protein/energy intake, and the resulting imbalances in micronutrients, energy reserves and protein levels yield negative effects on their development and growth (Dipasquale et al., 2020). When children are malnourished, they tend to have lower academic performance and productive life, and they have higher odds of developing infectious and chronic diseases, than well-fed children (Ghimire et al., 2020). Importantly, depending on the undernutrition status, there could be various risks of death, mainly due to dysregulation in the immune system and infectious diseases (Ghimire et al., 2020). Thus, it is not surprising that SAM children have 9 to 11.6-times greater mortality rates than children under normal nourishment conditions (Bhadoria et al., 2017; Barba et al., 2020).

### Clinical consequences of SAM in relation to sepsis

SAM is a multidimensional disorder that is strongly associated to adverse socioeconomical environments, including poverty, social exclusion, families with 3 or more children, illiterate mothers, low dietary diversity, lack of hygiene, non-adequate toilet facilities, and the consumption of polluted water (Collins et al., 2006; Ghimire et al., 2020). Therefore, it is expected that these children are highly exposed to pathogens, particularly to enterobacterial microorganisms. Also, as the acute malnutrition evolves into a severe and even a normalized state for the children, their physiology adapts to limited nutrient intake, creating an austere distribution of energy and diminishing the capacity of children to resolve stressful conditions and infections (Collins et al., 2006). Indeed, in SAM children, both gastroenteritis and acute respiratory infections are expected diseases (Collins et al., 2006), and it is known that such infections commonly evolve into septicemia, especially by Gram negative bacteria like *Salmonella* spp., *Klebsiella pneumoniae* and *E. coli* (Page et al., 2013). Since relative abundance of the latter correlates with resistome composition, and antimicrobial resistance gene mobility until diversity threshold is reached during microbiome maturing, it is indeed worrisome if concurrent dysbiosis leaves antimicrobial resistance unchecked (Lebeaux et al., 2021). Even if these children do not manifest the traditional symptomatology of an infection, it is common to treat them with broad-spectrum antibiotics at the moment of their presentation at the hospital, in part because SAM children usually harbor bacterial overgrowth in the small intestine, and they might not have apparent signs of underlying bacteremia (Jones and Berkley, 2014). When possible, the preventive use of antibiotics such as co-trimoxazole, amoxicillin, ampicillin, and gentamycin, is encouraged in this population (Jones and Berkley, 2014; Richard et al., 2020). But in some cases, co-trimoxazole and amoxicillin are not useful, and this is due to the antibiotic resistance of pathogenic enterobacteria toward these drugs (Page et al., 2013). One report from 2017 in a Tanzanian hospital covering patients with a median age of 17 months old from the Victoria Lake zone, showed that unlike mild/moderate malnourished hospitalized children, the population with SAM had a significantly higher prevalence of bacteremia (Ahmed et al., 2017). Most of the isolates were Gram negative bacteria, especially *Pseudomonas* spp., and all of them were resistant to ampicillin, particularly *E. coli*, which showed the highest resistance rate for gentamicin and third generation cephalosporines across all the isolates (Ahmed et al., 2017).

As expected, the use of antibiotics is not always a possibility for SAM children. Thus, a direct secondary effect of the prevalence of pathogen infections is a constant state of inflammation, starting at the intestinal level, which could worsen the effects of malnourishment.

## Malnourished children are in a constant state of inflammation and have slower growth rates

### Immunopathology of environmental enteric dysfunction and low energy immunometabolism rewiring

Environmental enteric dysfunction (EED) and stunt growth are pervasive in SAM children, resulting from a two-hit phenomenon; insufficient tryptophan and choline induce inmunometabolic rewiring, preventing gain of immunotolerance, whereas constant PAMPs exposure and inflammation reinforce malnutrition and infection.

**Figure 1:**
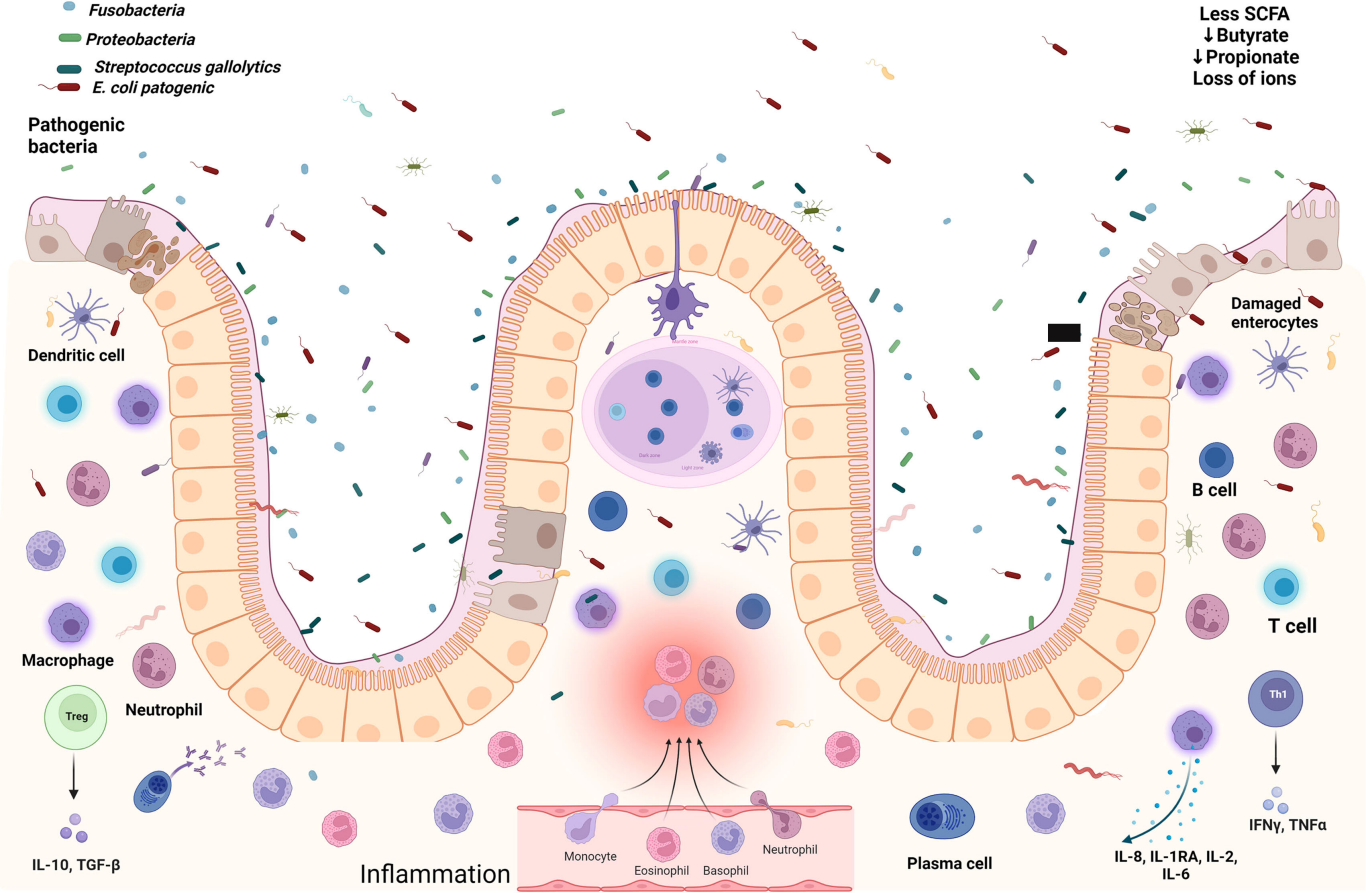
The gut microenvironment in severe acute malnourished children. Damaged enterocytes, several immune cells overwhelmed with cytokines, pathogenic bacteria, aerobic environment with less SCFAs, and loss of ions promoting gastroenteritis. Created with BioRender.com.

Under normal conditions and appropriate nutrient intake, the submucosal layer of the intestine is enriched with infiltrated Foxp3^+^ T regulatory (Treg) cells that maintain a homeostatic state through permanent communication with healthy microbiota, and together, both ensure a low inflammatory environment (Cosovanu and Neumann, 2020). In SAM children, this immune equilibrium is disrupted to favor Th1 immune pro-inflammatory conditions in the intestine, driving environmental enteropathy, **Figure 1**, especially in the small intestine (Bartels et al., 2019).

The persistence of inflammatory infiltrates, such as IFN-γ derived from Th1 lymphocytes, promotes crypt hyperplasia and villous atrophy, which could induce loss of the intestinal barrier to increase the permeability of the intraepithelial layer (Attia et al., 2016; Bartels et al., 2019). Additionally, the intestinal damage in SAM children enables the chronic exposure to bacterial products such as LPS, thus enhancing the state of systemic inflammation in a progressive positive cycle (Patterson et al., 2021). LPS, also known as endotoxin, is a highly immunogenic glycolipid molecule found on the outer membrane of Gram negative bacteria like *E. coli* (Simpson and Trent, 2019), whose presence in circulation has been linked to sepsis and to the release of high levels of pro-inflammatory cytokines by immune cells, and under high concentrations, LPS can induce the development of the systemic inflammatory response syndrome with a dose-dependent mortality of the patient (Seemann et al., 2017). In order for the host cell to recognize LPS, this lipid must bind to the LPS-binding protein (LBP) which allows the contact of LPS to a cell surface, where the Toll-like receptor (TLR) 4 specifically recognizes LPS and, as a result, an intracellular cascade driven by intermediate molecules such as MyD88 and NF-κB ensures the production of pro-inflammatory cytokines (Roger et al., 2009), yielding strong Th1 immune responses (McAleer and Vella, 2008). The LPS found in *E. coli* has demonstrated the ability to induce systemic hypoglycemia, and 2 hours and 4 hours after its inoculation, this lipid increases the serum levels of the pro-inflammatory cytokines tumor necrosis factor (TNF)-α and IFN-γ 4,500% and 25,000% more than non-inoculated controls, respectively (Seemann et al., 2017). Overall, the pro-inflammatory events triggered by the upregulation of Th1-related cytokines in SAM follow the same trend seen in other cell-mediated inflammatory diseases of the intestine, including inflammatory bowel disease (IBD) (Attia et al., 2016). Interestingly, at the histological and immune levels, SAM changes resemble those seen in IBD, Crohn’s disease and in food allergy, and just like in patients suffering those conditions, the use of hypoallergenic elemental formula composed of single amino acids has been demonstrated to be tolerated in SAM children as well, who additionally gain weight upon this approach (Bartels et al., 2019). With nutritional rehabilitation, the total leukocyte counts in these children increase as well (Njunge et al., 2020). However, the metabolic profile of SAM children is characterized by low serum concentrations of essential amino acids, and biogenic amines including kynurenine-tryptophan-nicotinamide metabolic pathway, a central hub to a plethora of adaptative responses besides to energy, which are supported by mitochondrial NAD^+^. Carnitine, as well as, ω-3, ω-6 polyunsaturated fatty acids deficiency, might be cause and consequence explaining that even after stabilization of their nutritional status, and though growth recovery could be achieved, the incidence and mortality by infectious diseases are still maintained (Patterson et al., 2021).

### Biomarkers of the severity of inflammation in severe acute malnutrition

Certain biomarkers can be measured to evaluate the severity of inflammation in SAM, such as the intestinal inflammation marker fecal calprotectin, which is an acute phase protein upregulated in these children (Bartels et al., 2019) that is significantly associated with both the presence of multiple intestinal pathogens and with death (Attia et al., 2016). In a recent multi-center, randomized, controlled case-control study in SAM children from Kenya and Malawi, it was reported that circulating proteomic and metabolomic profiles were different between children who survived from those who died, the latter being enriched in calprotectin and C-reactive protein (CRP), and in the inflammatory intermediates interleukin (IL)-8 and TNF-α (Njunge et al., 2020). Further systemic inflammation markers, including IL-1RA, IL-2, IL-6, granulocyte colony-stimulating factor (GCSF), TNF-α and TNF-β, have been reported to be upregulated in children dying due to SAM, irrespectively of their age and sex, but without predicting the presence of a specific intestinal pathogen (Attia et al., 2016). Additional indicators related with the inflammatory status in SAM have been reported as well. These include the intestinal mucosa integrity markers fecal α_1_-antitrypsin, reflecting intestinal protein loss; plasma intestinal fatty acid-binding protein (IFABP), which indicates enterocyte lysis; and plasma IgG anti-endotoxin, which implies gut bacteria translocation, all of which are upregulated as well under SAM conditions (Bartels et al., 2019).

In an inverse way, the plasmatic growth factors insulin-like growth factor (IGF)-1 and its main ligand, IGF-binding protein 3 (IGFBP3), are downregulated in SAM children (Timothy, 2011; Bartels et al., 2019), and since IGF-1 is an anti-apoptotic peptide associated with mitosis and bone growth (Wen et al., 2022), it is expected that their low circulation levels reflect the slow growth rate seen in SAM. Certainly, a further secondary effect of systemic inflammation in SAM children is a negative linear growth (Njunge et al., 2020). This statement is supported by a Kenyan study with HIV-positive SAM children responding to feeding therapy, which showed an average height increase of 0.34 mm/day over 60 days of follow-up, but demonstrated a negative correlation between the systemic inflammatory markers IL-2, IL-17α, and LBP with their growth (Njunge et al., 2020). Additionally, Maleta K. et al. reported, in 2021, a comparison between 716 Malawian and 80 Finnish children and found that at 18 months old, Malawian children had almost half the concentration of IGF-1 of that found in Finns (Maleta et al., 2021). They also reported a strong relationship of low plasmatic IGF-1 levels with the systemic inflammatory marker CRP in a dose-dependent way, and with intestinal infection by *Campylobacter* or *Shigella* bacteria (Maleta et al., 2021). Despite the fact that it has not been clinically evaluated in SAM children, an experimental work performed in dogs inoculated with *E. coli*-derived LPS has shown an important decrease in IGF-1 levels, as well as in the anti-diabetic hormone adiponectin (Tvarijonaviciute et al., 2011), suggesting that infection with this bacterium potentially could limit growth while promoting diabetes in affected children.

Although low concentrations of IGF-1 are seen in SAM children, its positive regulator growth hormone (GH) can be found upregulated. Briefly, IGF-1 is synthesized in the liver due to stimulation by GH (Chia, 2014). The persistence of GH prevents glucose uptake by peripheral cells such as myocytes and leukocytes to ensure its internalization in brain, erythrocytes and kidney, but such a role of GH as a glucose modulator is independent of its pro-IGF-1 function (Freemark, 2015). As will be explained later, low levels of IGF-1 at early ages are related to the development of non-communicable diseases such as diabetes (Bourdon et al., 2019), an effect that could be promoted in parallel by high concentrations of the diabetogenic hormone GH in SAM children (Kim and Park, 2017).

IGF-1 has additional roles other than the mere promotion of cell growth. Hunninghake G. et al. proved, in mice undergoing experimental sepsis, an inverse correlation between circulating concentrations of IGF-1 with bacterial translocation and migration to liver (Hunninghake et al., 2010). Contrarily, when IGF-1 was administered to mice, bacterial translocation was reduced, and apoptosis of cecum epithelial cells previously seen in sepsis was prevented, thus demonstrating that IGF-1 also promotes the stability and survival of gastrointestinal cells (Hunninghake et al., 2010). Such living cells act as docking sites, allowing symbiotic commensal bacteria to attach, which, in turn, ferments fiber and synthesizes short-chain fatty acids (SCFAs). SCFAs dynamically stimulate the production of both IGF-1 at systemic and bone marrow levels, and of the master regulator of osteoclastogenesis RANKL to modulate growth in the bone (Yan et al., 2016; Yan and Charles, 2018). Indeed, in 2021, a report from Indonesia showed that the overall SCFA concentration in stool samples were significantly lower for butyrate and propionate in malnourished children versus their healthy counterparts, and contrarily, a significant decrease in height was found in undernourished conditions (Kamil et al., 2021).

### *Escherichia coli* pathotypes mechanisms directly causing malnutrition preparing the assault of intestinal niche

In addition to indirect effects pertaining to a perturbation of a healthy microbiome, a link has been found between Enterotoxigenic *Escherichia coli* (ETEC) LT toxin ability to induce cAMP, the activation of NFκ-beta signaling pathway and a reduced transcription of ascorbic acid transporters (SLC23A1, SLC23A2) and/or by a related mechanism which inhibits thiamine uptake (SLC19A2, SLC18A3) into intestinal host cells. Depletion of enzyme cofactors and antioxidants may represent a common competing strategy on persistent intestinal infection pathogenesis, since only functional 3TSS EPEC espF, espG exerts control over hGRHPR and hSVCT1 controlling ascorbic acid uptake whereas espF and espH inhibits thiamine transport *via* transcriptional control as well.

Despite being a much less explored avenue transiting the interaction between enteropathogenic *Escherichia coli*, microbiome and host health, the co-metabolism of tryptophan and specifically, key ability to modulate serotonin (5-HT) concentration in extraintestinal compartments, proves that direct impacts of infection over mineral metabolism are plausible, thus opening several possibilities around pathogenic interaction. Whereas complexity is increasingly acknowledged; hypothetically, redox potential might be playing the melodic part since, at least *in vitro*, ST toxin from ETEC responds by reconstituting its binding capacity to zinc (Zn) or Iron (Fe) in parallel to aerobic-anaerobic conditions. Although the homeostatic detoxification role *in vivo* is still debated, it is straightforward that Zn deficiency translates into a more virulent EAEC gene expression profile and sub optimal immune performance, whereas Zn overload in *Escherichia coli* inhibits iron -sulfur cluster biogenesis, not to mention blockade of SOS hypermutator response and horizontal gene transfer inhibition at lower Zn concentrations, preventing EPEC from developing antibiotic resistance emergence *in vivo* (**Table 1**).

**Table 1:** Growth and inflammation caused by metabolites from human gut microbiota.

Metabolite	Models	Effects
Choline	Serum from children	Choline is associated to child growth and development by the essential role in the linear growth bone, Cell membrane formation, lipid metabolism, pulmonary function, neurotransmission and central nervous system development and gut immunity (Attia et al., 2016).
Tryptophan	Host and microbial metabolism	Lower tryptophan levels and increased tryptophan related enzymes and downstream metabolites have been linked to increased metabolic inflammation and fibrosis (Teunis et al., 2022).
	Mouse model	Children with severe malnutrition have lower serum tryptophan levels. Then, the effects of modulating the tryptophan-nicotinamide pathway are mediated through sirtuin1, which is associated hepatic metabolic dysfunction (Hu et al., 2020).
	Metabolomics assays	In *E. coli* production and release of indole-based metabolites or nucleotides has been linked to cellular, tissue, and host well-being (van der Hooft et al., 2019).
Protein or zinc	Mouse model	Protein or zinc deficiency, profoundly alters weight loss, pathogen shedding, and biomarkers of intestinal disruption in EAEC, ETEC, *Shigella*, and *Campylobacter* infections in mouse model (Bolick et al., 2014; Guerrant et al., 2021).
Carnitine	Serum from children	Secondary carnitine deficiency in children with environmental enteric dysfunction (small intestine inflammation and abnormal gut permeability), with altered metabolism associated with abnormal gut microbiome (Semba et al., 2017).
Gluconate, galacturonic and glucuronic acids	Gene expression assay	Catabolism of galacturonic and glucuronic acids is important for colonization by EHEC, while commensal *E. coli* use gluconate but not galacturonic and glucuronic acids (Jimenez et al., 2020).
Short-chain fatty acids, methylamines, and indoles	Human gut microbiota	These gut microbial metabolites in human contribute to host health and disease (Abdul Rahim et al., 2019).
Phosphatidylcholine	Bacterial and mouse model	The incorporation of phosphatidylcholine into *E. coli* membrane enhances the ability of bacterial cells to resist the AMPs cecropin P1 and indolicidin, changes hybridizing patterns to the periplasmic proteins and lipopolysaccharides and cell morphological character and decreases bacterial attraction to mouse macrophages *in vivo* (Chen et al., 2009).
Arginic Acid	Metabolomics assays	Arginic Acid promotes oxidative damage, and the molecule was found among a panel of metabolites associated with chronic kidney disease (van der Hooft et al., 2019).

When SAM develops, the delicate equilibrium between the maintenance of a healthy growth rate, a low inflammatory microenvironment and microbiota homeostasis is disrupted. If the normal microbiota is altered, dysbiosis is then triggered to potentially worsen the prognosis of SAM children.

*E. coli* is a rod-shaped Gram negative, facultative anaerobic commensal bacterium that trains the immune system (Fábrega et al., 2016), and which promotes a suitable microenvironment for other healthy anaerobic bacteria (Martinson and Walk, 2020). However, *E. coli* also possess an open genome, and may act as generalist, colonizing new niches and exploiting intestinal inflammation oxygenic conditions. The main pathotypes of *E. coli*, named EAEC, EPEC, ETEC and EHEC, are among the key microorganisms responsible for gastroenteritis diseases in low- and middle-income countries, especially in overcrowded human communities with either none or limited access to potable water and adequate water services.

Its ability to establish there is dependent on the availability of specific carbohydrates in the mucus layer of the gut epithelium, including fucose, hexuronates, arabinose, lactose, sucrose, mannose, gluconate, ribose, *N*-acetylglucosamine, *N*-acetylneuraminate, and *N*-acetylgalactosamine (Martinson and Walk, 2020); however, although the Genome Wide Association indicates sialic acid uptake by *nan9* gene acetyl esterase clusters to human, precise outcome of interaction seems to be influenced by intestinal membrane integrity (Fábrega et al., 2016) and anaerobic comensal crosstalk delicate equilibrium.

## The malnutrition-dysbiosis link: Causes and consequences

**Figure 2:**
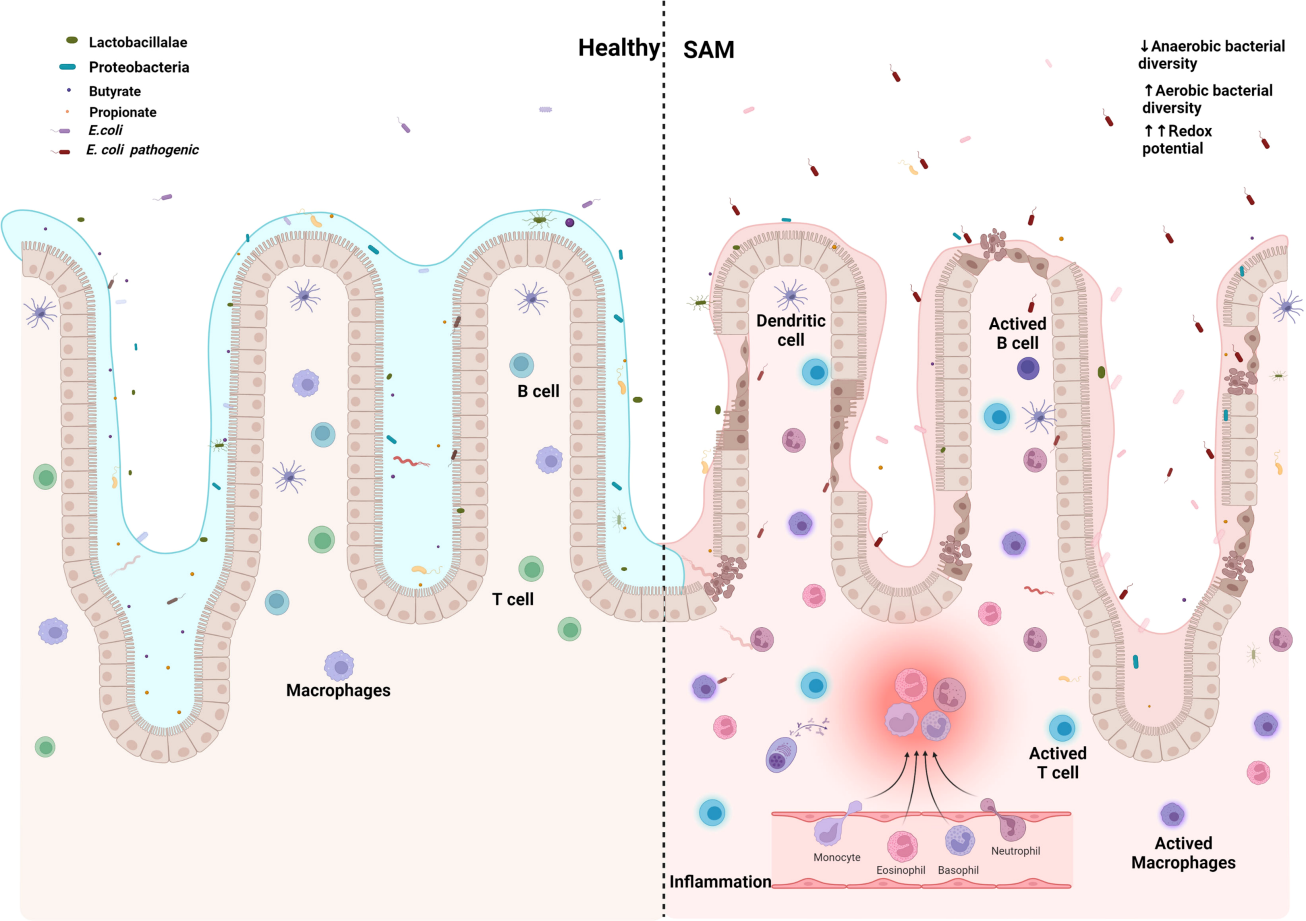
*Escherichia coli* establishes and maintains the damaged gut microenvironment in severe acute malnutrition. Created with BioRender.com.

The continuous presence of microbial pathogens, together with the lack of appropriate nutrition intake and the dysbiosis resulting from bacterial overgrowth in the intestine, results in the persistence of a cycle of gastrointestinal pathology and inflammation. In addition, the microbiota dysregulation modifies the production of specific metabolites, and limits the re-establishment of the healthy local flora, **Figure 2**.

In substitution of symbiotic gut microbiota with *E. coli* common pathotypes in developing countries, EPEC, ETEC, and EAEC have direct and undirect effects over Z - growth score and intestinal health. In mice, EPEC gravelly impacts TCA cycle, reducing concentration of metabolites, particularly of pantothenate precursor of Coenzyme A, necessary for β-oxidation, partially recreating SAM energy metabolome, showing increased ω-oxidation, followed by increased production of taurine, because of carnitine deficiency, which is in turn also associated with the increased intestinal permeability of EED (**Table 2**).

**Table 2:** Growth and inflammation caused by *Escherichia coli* pathotype.

*Escherichia coli* pathotype	Models	Effects
Enteropathogenic *Escherichia coli* (EPEC)	Mouse model	Colonization of EPEC, promotes the development of osteolysis by increasing the secretion of peripheral 5-hydroxytryptamine from the colon (Xue et al., 2022).
	Mouse model	The evaluation of an EPEC infection model, showing how disruption of bfp function does not impair, and may even worsen diarrhea, colonization, and intestinal disruption and inflammation (Ledwaba et al., 2020).
Enterotoxigenic *Escherichia coli* (ETEC)	Transcriptomics and metabolomics assays (Luria Bertani broth)	ETEC or *Escherichia coli, d*uring progression from logarithmic to early stationary phase is involved in the degradation of neurotransmitters aminobutyrate (GABA) and precursors of 5-hydroxytryptamine (serotonin) (Joffré et al., 2022).
Enterohemorrhagic *Escherichia coli* (EHEC)	Mouse model	The cysteine-responsive regulator, CutR, converges with the YhaO serine import pump and the fatty acid metabolism regulator FadR to optimally control virulence expression in EHEC (Pifer et al., 2018).
	Mouse model	In EHEC a screen identified transcriptional regulators that respond to fluctuations in amino and fatty acids as playing an important role in virulence gene expression both *in vitro* and during mammalian infection (Pifer et al., 2018).
	Infection model (HeLa or Caco-2 cells)	EHEC interacts with arachidonic acid, a host derived and dietary polyunsaturated fatty acid, can impact the outcome of enteric pathogen (Ellermann et al., 2021).
Adherent and invasive *E. coli* (AIEC)	Mouse model	Mucosal metabolites associated with health and inflammation for growth and virulence (Zhang et al., 2017).

Bacteremia is a common condition in SAM children, affecting up to 17% of them (Patterson et al., 2021). In fact, a meta-analysis covering 140,148 SAM children from 54 African studies has demonstrated that HIV-infected children, unlike their non-HIV-infected counterparts, showed worse prognosis, and in fact their recovery rate was reduced by 81%, which could in part be attributable to concomitant infections such as bacteremia (Desyibelew et al., 2020). One cohort study in Uganda enrolled, from September, 2003 to December, 2004, a total of 450 SAM children infected by HIV, and found that among other pathogens and irrespectively of the HIV status, blood specimens demonstrated that these children were mainly infected by the Gram positive bacteria *Staphylococcus aureus* and *Streptococcus pneumoniae*, or by the Gram negative pathogens *S. typhimurium* and *S*. *enteritidis* (Bachou et al., 2006). An additional report performed between October, 2007 to July, 2008 with 311 SAM children hospitalized in an intensive care unit in Niger, reported bacteremia in 51 of these patients, mainly by *Salmonella* spp., *S. aureus*, *K. pneumoniae* and *E. coli* (Page et al., 2013).

On the other hand, it is expected that due to the lack of hygiene SAM children are exposed to, they are affected by gastroenteritis. Diarrhea, including acute infection gastroenteritis, environmental enteric dysfunction, osmotic diarrhea with dehydration, and HIV enteropathy, has been reported in up to 47-67% of SAM cases, and together they are related to inflammatory states, malabsorption, nutrient loss, anorexia, cachexia and ultimately high mortality rates (Attia et al., 2016; Alaaraj et al., 2021; Olupot-Olupot et al., 2021). When the dehydration induces advanced shock, which could be achieved by the presence of ≥3 watery stools/day, up to 82% of SAM children experience death by day 28 (Olupot-Olupot et al., 2021).

### Occurrence of dysbiosis among SAM children and pathogen prevalence

In EED, like in other enteritis, a significative reduction of bacterial biodiversity parallels the weakening of several functions including colonocyte nutrition, immune training, and resistance to infection of several pathogens mediated by SCFA metabolites.

A Nigerian work has shown diarrhea in 53% of tested SAM children, of whom 37% were positive for an enteric bacteria such as *Salmonella* spp. and *Campylobacter jejuni* (Page et al., 2013). In a 2013 cohort in Malawi enrolling 79 children with SAM, it was reported that most of them were positive for intestinal pathogens including *Campylobacter jejuni*, enterotoxigenic *E. coli* (ETEC), *Clostridioides difficile*, *Salmonella* spp., *Shigella* spp., *Giardia lamblia*, *Cryptosporidium parvum*, and *Entamoeba histolytica*, and, importantly, over 40% of the children had at least 2 of said pathogens (Attia et al., 2016). Not only ETEC, but also enteropathogenic and enteroaggregative *E. coli* (EPEC and EAEC, respectively), have been found in the feces of SAM children with diarrhea (Tickell et al., 2020). Certainly, the Global Enteric Multicenter Study (GEMS) cohort has reported, in 8,182 children with moderate-to-severe diarrhea versus 11,590 controls, all from Bangladesh, India, Kenya, Mali, Mozambique, Pakistan, and Gambia, that the presence of acute malnutrition increases the odds of diarrhea for typical EPEC among children 6-11 months of age, and for ETEC among children 12-23 months old (Tickell et al., 2020). Additionally, an interaction was reported between *Shigella* spp., norovirus and ETEC in children 6-11 months old, and of *Vibrio* spp., norovirus and EPEC in children 12-24 months old, and, importantly, ETEC and both typical and atypical EPEC were strongly associated with case fatality rates (Tickell et al., 2020).

The rise in these species of bacteria implies that the healthy microbiota is modified or diminished, and, as a consequence, dysbiosis occurs. Importantly, the fact that therapeutic diet and amoxicillin fail in some cases to prevent refractory cases, or even death by enteric sepsis, suggests an irreversible disruption of the gut microbiota (Tidjani Alou et al., 2017). Under healthy circumstances, symbiotic anaerobic bacteria belonging to the *Lactobacillalae* and *Proteobacteria* taxa in the gut block pathogen colonization through the competition of nutrients, by direct killing, by the stimulation of immune responses, and by the production of inhibitory metabolites such as SCFAs, which are effective against *C. difficile* and pathogenic *E. coli* strains (Pickard et al., 2017). By using metagenomic approaches, it has been reported that malnourished children harbor a decreased anaerobic bacterial diversity in the gut, consequently revealing an unhealthy status of the intestinal microbiota and a high redox potential, as well as downregulation of the adiposity- and weight gain-related bacteria *Methanobrevibacter smithii*, and enrichment in Fusobacteria, Proteobacteria and *Streptococcus gallolytics*, the three potentially pathogenic bacteria species (Tidjani Alou et al., 2017). Concerning *M. smithii*, it is a bacterium known to deplete the presence of fermentative dihydrogen in the gut, improving the oxidation of substrates for energy harvesting, the production of butyrate and acetate, both being SCFAs, and thus promoting gut homeostasis (Camara et al., 2021). A case-control study in Mali compared the feces from 143 malnourished children against 110 healthy children, revealing that *M. smithii*, was significantly downregulated in stool samples from SAM children even after the administration of therapeutic diet (Camara et al., 2021). An additional Ugandan study reported that dying SAM children had a poor gut microbiota diversity, with a significant presence of Proteobacteria and *Enterobacteriaceae*, and reduction in the SCFAs-producing *Bacteroidetes* and *Firmicutes*, and importantly, the marker of gut inflammation fecal calprotectin was upregulated in these children (Calder et al., 2021).

Both inflammation in SAM and intestinal function are regulated by SCFAs. Starch and dietary fiber, both of which are not digested in the upper gastrointestinal tract, are anaerobically fermented mainly through glycolysis by resident microbiota in the gut, generating SCFAs (Parada Venegas et al., 2019; Calder et al., 2021). Indeed, SCFAs such as butyrate, acetate and propionate, are secondary products released as by-products of fermentation by healthy microbiota, microorganisms including *Lactobacillus reuteri*, *Faecalibacterium prausnitzii*, *Bifidobacterium longum*, *Akkermansia muciniphila*, *Roseburia* spp., and *Clostridium* cluster XIVa species (Morrison and Preston, 2016; Yan et al., 2016), and among other roles, they improve energy yield, maintain the mucosal integrity in the gut, regulate the colonic pH, prevent the accumulation of potentially pathogenic organisms, and produce vitamins (Calder et al., 2021). Particularly, butyrate is an anti-inflammatory lipid that acts as a primary energetic source for the local gastrointestinal epithelial cells (Attia et al., 2016; Parada Venegas et al., 2019), and propionate, the most abundant SCFA, is a specific smooth muscle relaxer and vasodilator acting on mesenteric small arteries to allow blood flow and oxygen into the colon (Calder et al., 2021). Moreover, the metabolism of these SCFAs by epithelial cells that internalize them requires the consumption of O_2_, which stabilizes the hypoxia-inducible factor and regulates epithelial gene expression associated with lipid metabolism and with blockade of proliferation in stem cells (Parada Venegas et al., 2019). Since SCFAs block LPS-induced NF-κB activation, it is not surprising that they possess anti-inflammatory properties, including both the expression of IL-10 (Parada Venegas et al., 2019), and the upregulation of colonic Foxp3^+^ Treg cells (Kim et al., 2014).

Under SAM conditions, relatively immature microbiota has been reported (Calder et al., 2021), resulting in reduced levels of fecal propionate and butyrate, which is related to diarrhea and death in affected children (Attia et al., 2016; Calder et al., 2021). Between 2003 and 2005, 175 severely malnourished children with cholera from Bangladesh were found to have SCFA concentration of 4.7 mmol/kg in feces at day 0, but increased upon 7 days of treatment with ampicillin plus gentamycin and erythromycin to up to 51.7-95.0 mmol/kg at day 28 after admission, making levels comparable to those seen in healthy children having 102.0 mmol/kg of SCFAs (Monira et al., 2010). These children also had a shift in their bacterial population, from an enrichment in *Enterobacteriaceae* and downregulation in *Bifidobacteria* at day 0 of treatment, to an upregulation over time with *Bifidobacterium*, *Bacteroides* and *Lactobacillus*, reflecting the increase in the SCFAs in their feces (Monira et al., 2010). The low presence of SCFAs, in turn, allows the colonization of pathogenic *E. coli* bacteria (Pickard et al., 2017). Unlike probiotic and even commensal strains, multi-drug resistant (MDR) *E. coli* strains show an improved capacity to adhere and invade the mucosal layer of the gut, thanks to their type I fimbriae, thereby limiting the capacity of the healthy microbiota to interact and promote intestinal homeostasis (Sarkar et al., 2018).

As suggested above, the acquisition of specific pathogens such as *E. coli*, belonging to the phyla Proteobacteria, could displace the presence of symbiotic bacteria from healthy gut microbiota. Although *E. coli* is present in over 90% of subjects as a non-pathogenic bacterium in their normal microbiome, arising even as one of the first bacteria colonizing the gut of neonates after birth (Martinson and Walk, 2020), exposure to pathogenic strains such as EPEC, EAEC and ETEC prompts the development of dysbiosis in SAM. Importantly, with the use of animal models of malnutrition, an increased susceptibility to ETEC has been discovered, due to malabsorption, intestinal damage, nutrient loss, and overall disruption of the immune response and of its energetic depots (Patterson et al., 2021). Additionally, it is known that gastrointestinal infection by the enterohemorrhagic *E. coli* (EHEC) strain O157:H7, which produces Shiga toxins, is associated with the development of hemolytic-uremic syndrome (HUS), and, at later ages, with the onset of insulin-dependent diabetes due to β-cell mass loss at pancreatic islets, with secondary insulin deficiency (Suri et al., 2009; Lim et al., 2010). Additionally, a higher microbiome content of *E. coli* has been related to long-term conditions such as obesity, the progression of non-alcoholic steatohepatitis, and even to the formation of amyloid plaques in Alzheimer’s disease (Muscogiuri et al., 2019; Vamanu and Rai, 2021). Thus, the potential effects of *E. coli* strains for the promotion of chronic conditions in surviving SAM children urges a further deeper analysis.

## Isolation of *E.coli* from unhealthy water sources and soils

Although the primary habitat of this bacterium is the gut of warm-blooded animals, other niches in which it establishes interactions with bacteria are soil, sediments and water microbiomes, making their functions as so-called “gene mixing vessels” and/or “distribution highways” for antimicrobial resistance (Hayashi et al., 2019; Pärnänen et al., 2019; Kunhikannan et al., 2021) a major concern, given the level of exposure to fecal contaminated water.

As mentioned above, frequent environmental exposure, jointly with EED, and stunt status modify risks related to inflammation. In the Sub-Saharan region of Africa, where merely 56% of the population are able to consume ≥20 L water per daily, and other deprived areas worldwide, multiple sources, of *E. coli*, includes hands, soil, flies, food, and polluted water (Navab-Daneshmand et al., 2018), are also sources of antimicrobial resistance.

Previous studies have demonstrated the presence of *E. coli* in water sources destined for human consumption, and in both farms and soils where open defecation and inadequate disposal of wastewater and of animal feces are found (Harada et al., 2018). A report from Burkina Faso confirmed *E. coli* values in the range of 50-15,000 MPN/100 mL after collecting 38 samples from surface water depots between August, 2018 to April, 2019, and indicated a strong correlation between *E. coli* levels and the incidence of diarrheal diseases corresponding to seasons with high pluvial precipitation due to a boost in the use of low-grade quality water by the population (Hayashi et al., 2019). In Kenya, after 7 months of close monitoring, Nowicki S. et al. reported that in 44 sites coming from 9 community-managed water supply systems, which were obtained from boreholes or reservoirs and acted as the main or alternative source of drinking water, a median *E. coli* concentration of 1-920 MPN/100 mL (Nowicki et al., 2021) was found. Importantly, the authors performed a genomic classification on the samples and found >80 virulence genes in 4 isolates, as well as several EPEC-related virulence genes in 8 isolates, which is relevant since virulence genes are found more often when the strains are collected from human samples than when they are found in wild external sources (Nowicki et al., 2021).

As expected, the different pathotypes of *E. coli* have been specifically searched and reported in water depots and soil. Harada H. et al. analyzed 621 *E. coli* isolates recovered during 2014 from water sources in Bangladesh and found in 18.6% of the samples collected from sanitary wastewater depots the presence of *Stlb*-positive ETEC (Nowicki et al., 2021). An additional survey exhibited an even higher prevalence of ETEC across the tested samples. Indeed, Titilawo Y. et al. showed that among 300 of the *E. coli* isolates recovered from 10 different rivers used for livelihood, domestic and even recreational purposes, 45% of them were *lt*-positive ETEC (Titilawo et al., 2015). Furthermore, by collecting water samples between 2011 and 2012 in Pakistan, Shah M.S. et al. revealed that the flooding events occurring in 2010 were able to mobilize pathogenic *E. coli* strains into water sources for human consumption, explaining the acute diarrhea events that caused 13% of the 5.3 million medical consultations following the flood (Shah et al., 2016). The authors reported that 33% of the water samples were contaminated by pathogenic *E. coli* strains, of which 50% were ETEC and 29% were EPEC, and importantly, 97%, 91% and 70% of all the isolates were resistant against tetracycline, ciprofloxacin, and both cefotaxime and amoxicillin/clavulanate, respectively (Colavecchio et al., 2017).

AMR *E. coli* strains are able to share their resistant genes across bacteria in the microenvironment. This event is dramatically relevant in conditions such as SAM, where the diversity of bacteria is reduced to prompt the persistence of pathogenic and pro-inflammatory bacteria.

## Antimicrobial resistant *E. coli* and horizontal gene transference

Horizontal gene transference is concomitant to *E. coli* colonization and intestinal niche invasion quorum sensing guided process. Antimicrobial resistance and virulence factors are among the genes found in gut and environment.

**Figure 3:**
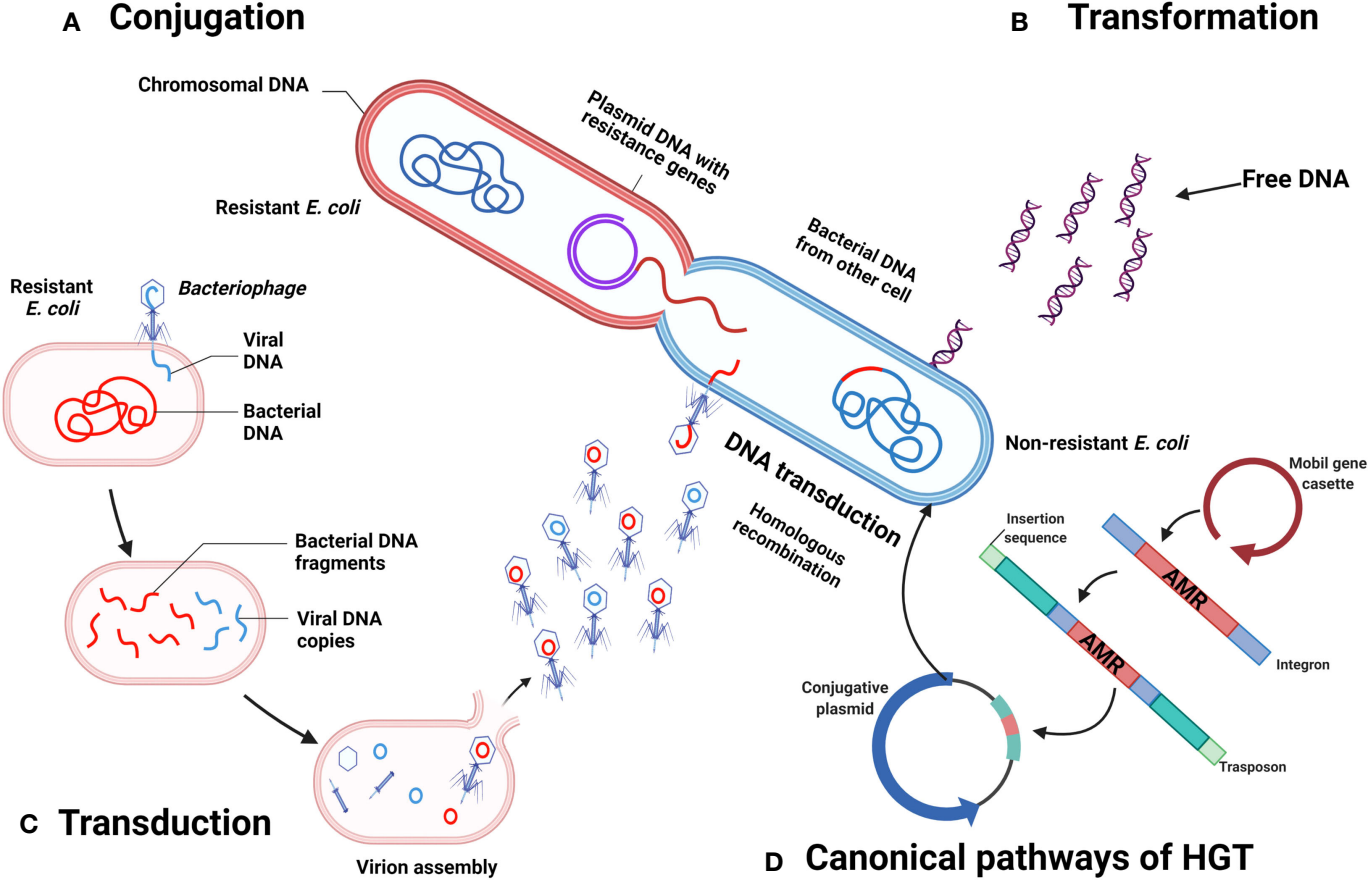
Horizontal transference of antimicrobial resistance genes by *E. coli*. **(A)** Conjugation. **(B)** Transformation. **(C)** Transduction. **(D)** Canonical pathways of HGT. Created with BioRender.com

The genome of *E. coli* is composed of nearly 2,000 genes, but almost half of it is dedicated to adaptive routes (Nowicki et al., 2021). The capacity of *E. coli* to persist over time relies on its ability of rapid evolution, which could be attributed to both mutation and recombination (Frazão et al., 2019). In the latter, horizontal gene transference (HGT) is considered a prominent feature for *E. coli*, which takes advantage of the gut microbiota, bacteriophages included, to allow a dynamic gene transference between resident bacteria and itself, **Figure 3** (Frazão et al., 2019). The recombinant process of *E. coli* is so important, that the classification of its pathotypes is established according to the presence of 14 virulence genes, including *afa/dra*, *aggR*, *daaE*, *eaeA*, *ipaH*, *iutA*, *kpsMT II*, *Ltl*, *papA*, *papC*, *sfa/foc*, *Stlb*, *stx1*, *and stx2* (Harada et al., 2018).

It is expected that in newborns, a colonization of *E. coli* should take place in the gut right after birth. This is due to exposure to the maternal vaginal and fecal microbiota at the moment of giving birth, and by breastfeeding, making these events a selection process by which the first bacteria colonizing the gut are capable of blocking the installment of potentially pathogenic bacteria (Ducluzeau, 1993; Hetzer et al., 2019). However, exposing the infants to antibiotics at an early stage of life may result in a disequilibrium of the colonizing bacteria, allowing the pathogenic bacteria to cause a variability in the normal colonization pattern (Könönen, 2000), including the installment of pathogenic *E. coli*, which may already carry AMR genes to commercially available antibiotics. Importantly, the increased resistance of *E. coli* causing common neonatal infections is a worldwide health concern, with some strains resistant to amoxicillin, cefuroxime, ceftriaxone, amoxicillin/clavulanic acid, cefoperazone-sulbactam, ceftazidime, gentamicin, ciprofloxacin, sulfonamide and meropenem, making MDR *E. coli* a problem within hospitals, neonatal wards, and health personnel (Könönen, 2000).

Briefly, there are 4 major resistance mechanisms in bacteria, which are: a) limiting the uptake of drugs by reducing or modifying the membrane permeability, modification of cell wall proteins, or secretion of biofilms; b) by efflux bombs responsible for removing toxic molecules from the organism; c) enzymatic activity, which is responsible of alteration or inactivation of drugs, many of which could be acquired by transfer of genes, i.e. resistance plasmids by HGT; and d) mutations or modification of antibiotic target sites. Regarding HGT, the common mechanisms in bacteria involve transformation, conjugation and transduction by phages. For a deeper explanation of these events, the reader is invited to refer to Thomas & Nielsen (Thomas and Nielsen, 2005).

The phageome could be seen as a reservoir of resistance genes. Previously, it was demonstrated in mice models that phages from antibiotic-treated bacteria with either ciprofloxacin or ampicillin, which eventually developed resistance toward the referred antibiotics, were responsible for the transference of these resistance genes to host bacteria from non-antibiotic treated mice (Modi et al., 2013). Interestingly, bacteriophages contribute to an increased spread of antibiotic resistance (Colavecchio et al., 2017). Phages which are also found in the gut of humans can also act as mediators for HGT within the human gut microbial ecosystem (Mirzaei and Maurice, 2017). In infants ranging from 0 to 3 years old, there is a dynamic microbiota fluctuation between bacterial and phages levels (Mirzaei and Maurice, 2017). It was previously suggested that prophages are the first type of phages that colonize the former bacteria in the gut of infants (Breitbart et al., 2008; Sharon et al., 2013; Mirzaei and Maurice, 2017). *Via* cross-kingdom effect, it is expected that with fluctuations in these dynamics, a high bacterial diversity can be present due to lytic infection (Weinbauer and Rassoulzadegan, 2004; Mirzaei and Maurice, 2017). However, these fluctuations are absent in adults (Mirzaei and Maurice, 2017).

In a study performed in mice continuously treated with antibiotics, it was found that they can affect the bacterial evolution in the gut due to a reduction in bacterial diversity, and thus limit the abundance of bacteria that carry lysogenic phages which are prevalent under normal conditions (Thompson et al., 2015; Kim and Bae, 2018). Another study demonstrated that in the mammalian gut, there is a continuous interplay between resident bacteria and newly incoming bacteria. In this sense, this interplay is by HGT, which plays a key factor in the evolution of *E. coli* (Frazão et al., 2019). Frazao et al. developed a gut-colonization model in mice, allowing a new *E. coli* strain to colonize the gut microbiota with resident *E. coli* strains, demonstrating that the rapid evolution of incoming *E. coli* is driven by phage-mediated HGT rather than through mutations (Frazão et al., 2019). In consequence, newly incoming *E. coli* acquired novel bacterial genes, conferring them metabolic fitness and adaptation to the environment, ultimately facilitating a stable colonization. Assuming a two-way avenue in host-microbiota, however, it is clear that in the case of ruminants, quorum sensing of metabolic cues, like vitamin K derivatives in EHEC, regulates in parallel toxin expression and dissemination of lambdoid phages bearing STX (Kijewski et al., 2020).

With this in perspective, in the monogastric digestive system, it is expected also that perturbations in the resident gut microbiota responsible for specialized tasks, could allow the colonization of other bacteria, such as pathogenic bacteria, and that they may encounter a fitness to establish themselves, and even share resistance genes, persist and adapt and, by HGT (Yaffe and Relman, 2020), increase the possibility of turning into MDR bacteria by plasmid exchange. For example, Stecher et al. demonstrated, in 2011, that under favorable conditions, such as inflammation of the gut, bacteria from the *Enterobacteaceae* group might exchange plasmids which harbor resistance genes. In this study, *Salmonella enterica* serovar Typhimurium (*S*. Tm) was able to transfer plasmid 2 (p2), a plasmid that belongs to a family that encodes for multiple antibiotic resistance genes, to resident *E. coli* in an HGT manner in the guts of mice, suggesting that infected patients could contribute to the spread of antibiotic resistance plasmids by changing the commensal bacteria genotypes (Stecher et al., 2012).

In turn, antibiotic treatments lead to an increased pool of genes encoded by gut phages. Should these genes confer resistance to the drug, then humans could be considered as suitable microenvironments where evolutionary mechanisms take place, turning resident bacteria into pathogens. In another study, using high-throughput chromosomal conformation capture, Yaffe and Relman compared stool samples taken 10 years apart from two individuals, and they could observe that the metagenomes encountered 12,252 accessory elements that were not annotated in the genome databases. They suggested that these accessory elements support the evidence of adaptive evolution in these bacteria (Yaffe and Relman, 2020). Plausibly, gene enrichment is determined by genome architecture; however, the outcome is also responsive to environmental cues. For instance, whereas *aggR*, a major regulator of EAEC virulence -and also the most associated factor to environmental enteric dysfunction and linear growth (Das et al., 2021), induces expression of virulence genes in response to oxygen concentration, it also causes a metabolic shift over gut and enhances transmission of virulence plasmids (Prieto et al., 2021). Speculatively, coupled regulation of *aggR* and N-HS (a thermoregulator silencer of AT rich or foreign DNA) *via* aar might have consequences beyond attachment timing; after all, H-NS regulates the expression of multidrug exporter genes (Nishino and Yamaguchi, 2004), and since plasmids coding H-NS like proteins as in IncX3 bearing Bla-NDM1, it improves plasmid stability and virulence (Liu et al., 2020).

Little information can be found in the literature about HGT by *E. coli* in SAM children. However, in these children, it is expected that the transfer mechanisms by MDR *E. coli* strains may exacerbate the spread of resistance across bacteria. For example, in hospitalized children with SAM in Niger, it was demonstrated that resident *E. coli* acquired β-lactam resistance during hospitalization (Woerther et al., 2011), which could be attributed to intrahospital acquisition of resistance genes by nosocomial bacteria. On the other hand, another study in Bangladesh covering non-malnourished children 10-24 months old, found by antimicrobial susceptibility assays that gut bacteria were already resistant to erythromycin, ampicillin, tetracycline, azithromycin, sulfamethoxazole-trimethoprim, cefixime and ceftriaxone. Some of the resident microflora carried gene cassettes, such as class 1 integron, *tetA, tetB, tetD, mphA, ereB, ermB*, β-lactamase genes, among others (Monira et al., 2010). With respect to resident *E. coli*, 11% of these bacteria possessed the gene encoding for Shiga toxin (*stx*)-2, and 22% possessed *eae*, which encodes an intimin adherence protein characteristic of EHEC (Monira et al., 2010). Surprisingly, 48% of the gut bacteria was found to carry a 140 MDa plasmid, which is associated with *Shigella flexneri* supporting the idea that HGT between phages, pathogenic and resident bacteria could play a role in MDR acquisition (Monira et al., 2017). This phenomenon deserves further exploration.

Additionally, the presence of virulence genes among *E. coli* isolations has been reported from water sources. Such genes are involved in multiple biological processes, including adherence, iron uptake, secretion, chemotaxis and motility, immune evasion, invasion and toxin production (Nowicki et al., 2021). In *E. coli* collected from water samples in Kenya, there have been reported up to 24 AMR genes, which allow the bacterium to survive β-lactams (*blaTEM-1*, *blaEC-5/8/13/15/18*, *blaCTX-M-14*), aminoglycosides (aa*dA1*, *aadA5*, *aac(3)-Iid*, *aph(6)-Id*, *aph(3”)-Ib*), erythromycin (*mph(A)*), sulphonamides (*sul1/2*), tetracycline (*tet(A)*, *tet(B)*), quaternary ammonium compounds (*qacEdelta1*), or trimethoprim (*dfrA1*, *dfrA14*, *dfrA17*, *dfrA5*, *dfrA5*) (Nowicki et al., 2021). However, whether these genes could be found in the resident microflora of people routinely using that water, and importantly, whether their acquisition confers a MDR phenotype as well, is still unknown and requires a deeper analysis.

## Conclusions

The most pervasive effect of SAM in children, EED and growth stunt are immunometabolism mediated, and are caused by the main pathotypes of *E. coli*, such as EAEC, EPEC, ETEC and EHEC. Dysbiotic niche promotes horizontal gene transference of antimicrobial resistance.

The HTG is dramatically relevant in conditions such as SAM, where the diversity of bacteria is reduced to prompt the persistence of pathogenic and pro-inflammatory bacteria. Concerning *E. coli* in SAM children, it is expected that the transfer mechanisms by MDR *E. coli* strains may exacerbate the spread of resistance across bacteria.

Now, environmental, social, and individual determinants of SAM and measures to avoid them are at least partially clarified.

## Author contributions

Conceptualization, RA-H and YL-V. Funding acquisition, YL-V, CS. Investigation, RA-H, CS, GC-R, SPdeL and YL-V. Methodology, RA-H, GC-R, SPdeL and YL-V. Resources, RA-H and YL-V. Writing – original draft, RA-H, GC-R, SPdeL and YL-V. Writing – review and editing, RA-H, CS, GC-R, SPdeL and YL-V. All authors have read and agreed to the published version of the manuscript. All authors contributed to the article and approved the submitted version.

## Funding

This work was supported by the grant Dirección General de Asuntos del Personal Académico-Programa de Apoyo a Proyectos de Investigación e Innovación Tecnológica (DGAPA PAPIIT) AV200320, IN210417 and IT202020 from the Universidad Nacional Autónoma de México (México), and by the Fundación Gonzalo Río Arronte Grant S.590.

## Acknowledgments

We thank Jesús Andrés Mejía Estrada and Claudia Ivette Rivas for helping with the references and drawing of pictures, respectively. We thank Susan Drier Jonas for help with editing the manuscript.

## Conflict of interest

The authors declare that the research was conducted in the absence of any commercial or financial relationships that could be construed as a potential conflict of interest.

## Publisher’s note

All claims expressed in this article are solely those of the authors and do not necessarily represent those of their affiliated organizations, or those of the publisher, the editors and the reviewers. Any product that may be evaluated in this article, or claim that may be made by its manufacturer, is not guaranteed or endorsed by the publisher.
